# Psychotropic pharmacogenetics in adult populations: From basic science to clinical trials and health economics – An evidence-based overview for decision makers

**DOI:** 10.1177/00048674251369625

**Published:** 2025-09-07

**Authors:** Trang TT Truong, Juliana Lago, Jennifer Neil, Fiona A Wilkes, Russell Barnes, Malcolm Hopwood, Ajeet B Singh

**Affiliations:** 1 Institute for Mental and Physical Health and Clinical Translation (IMPACT), School of Medicine, Deakin University, Melbourne, VIC, Australia; 2 Laboratorio de Genética Humana, Universidad de Los Andes, Bogotá, Colombia; 3 Department of General Practice, School of Public Health and Preventive Medicine, Monash University, Melbourne, VIC, Australia; 4 Academic Unit of Psychiatry and Addiction Medicine, The Australian National University School of Medicine and Psychology, Canberra Hospital, Canberra, ACT, Australia; 5 Department of Psychiatry, University of Melbourne, Melbourne, VIC, Australia

**Keywords:** Psychotropic pharmacogenetics, psychiatric care, treatment optimisation, health economics

## Abstract

Psychotropic pharmacogenetics (PGx) offers significant potential advancements in psychiatric care by optimising medication selection and dosing based on genetic factors. This perspective article highlights the clinical utility, health economic implications and implementation challenges of psychotropic PGx, proposing that its broader implementation could enhance patient outcomes and reduce healthcare costs. Landmark studies show that PGx-guided care results in fewer adverse drug reactions and improved medication efficacy, with substantial cost savings compared to traditional prescribing methods. However, implementation barriers persist, particularly in Australia, where knowledge gaps, limited clinical guidelines and funding constraints hinder adoption. Despite challenges such as industry bias and limited data on non-antidepressant psychotropics, robust clinical and economic evidence supports the expanded use of psychotropic PGx, with emerging combinatorial approaches offering promise for future psychiatric treatment.

## Introduction

Treatment resistance and adverse drug reactions (ADRs) represent major sources of healthcare burden in psychiatry. Treatment-resistant depression patients incur 2–6 times higher direct medical costs than treatment-responsive depression patients (Howes et al., 2022). Antipsychotics and antidepressants are among the top drug classes causing ADRs, together taking up to 86.1% of reported ADRs (Ambwani et al., 2021), while ADRs may cost the US healthcare system $30.1 billion annually (Sultana et al., 2013). In schizophrenia, early non-responders to antipsychotics require $1977 more in 12-week treatment costs than responders (Peng et al., 2011). Doctors and patients often struggle with the prolonged trial-and-error process of finding effective psychiatric medications, partly due to the weeks-long delay between initiating treatment and observing efficacy (Boyce et al., 2020; Malhi et al., 2018, 2021). This delay is thought to be linked to slower metabotropic processes, although the exact mechanism of psychotropic drug efficacy remains unclear, reflecting the complexity of the human brain (Malhi et al., 2021). Attempts to identify biomarkers based on psychotropic pharmacodynamics have been largely unsuccessful, as their mechanisms of action are not fully understood (Singh et al., 2014). However, epigenetic factors like gene silencing through methylation may help gauge individual pharmacodynamic profile (Hack et al., 2019). In contrast, significant advances in psychotropic dosing have emerged from a deeper understanding of pharmacokinetics, allowing clinicians to optimise dosing strategies. Pharmacogenetics (PGx) complements this progress by examining how genetic variations influence drug metabolism and interactions, enabling personalised treatment plans that adjust dosages to enhance efficacy and minimise adverse effects (Bousman et al., 2023; Singh et al., 2014). This perspective focuses on PGx applications in adult populations (⩾18 years), as paediatric PGx involves distinct developmental considerations beyond this scope.

## Empirical studies in PGx

In recent years, there has been a substantial increase in high-level evidence supporting the clinical utility of psychotropic PGx in improving the efficacy and tolerability of medications used in common psychiatric conditions, particularly major depressive disorder (MDD) (Amaro-Álvarez et al., 2024; Greden et al., 2019; Hernandez et al., 2024; Li et al., 2024a; Milosavljević et al., 2024; Santenna et al., 2024; Tesfamicael et al., 2024; Tiwari et al., 2022; Vilches et al., 2019). Three primary types of empirical studies deepen our understanding of PGx: genome-wide association studies (GWAS) (Hill et al., 2024; Li et al., 2024b), candidate gene association studies (CGAS) (Linskey et al., 2021; Luan et al., 2024) and randomised controlled trials (RCTs) comparing treatment as usual (TAU) with PGx-guided prescribing (Greden et al., 2019; Milosavljević et al., 2024; Santenna et al., 2024; Singh et al., 2014; Skokou et al., 2024; Tiwari et al., 2022).

These studies may be either industry-dependent or industry-independent, with the ideal scenario being a combination of both to mitigate the impact of commercial and publication biases on the integrity of the evidence (García-García et al., 2024; Medwid and Kim, 2024; *Saadullah Khani et al., 2024*; Tesfamicael et al., 2024).

For clinicians seeking to provide the best care and alleviate patient suffering, RCTs remain the gold standard for determining whether new approaches improve outcomes compared to TAU. However, before RCTs are conducted, GWAS and CGAS play a crucial role in identifying key genetic variants (polymorphisms) relevant to optimal medication dosing and selection – polymorphisms that affect medication response and occur frequently enough to have a significant impact on public health. The final and crucial layer of evidence is health economic utility of PGx – without economic feasibility, even the most promising innovations may fail to achieve widespread adoption. In the year since late 2023, health economic analyses from large, naturalistic, industry-independent studies have emerged, presenting a compelling case for the mass implementation of psychotropic PGx at a societal level (Basu et al., 2024; Frye and Nemeroff, 2024; Karamperis et al., 2021; Skokou et al., 2024).

## Clinical utility of psychotropic PGx

In 2023, a landmark RCT by Swen et al. assessing ADRs across both psychiatric and nonpsychiatric medications involving nearly 7000 adult individuals with psychiatric and nonpsychiatric conditions across eight European countries compared TAU to PGx-guided treatment for any new prescription of medications. This trial utilised a 12-gene panel focused primarily on pharmacokinetic polymorphisms based on the Dutch Pharmacogenetic Working Group (DPWG) guidelines, such as the phase I hepatic enzymes CYP2D6 and CYP2C19 (Swen et al., 2023). Notably, the Food and Drug Administration (FDA) and Clinical Pharmacogenetics Implementation Consortium (CPIC) guidelines align with DPWG recommendations for drug-gene interactions related to medication dosing and selection (Bousman et al., 2023; Brouwer et al., 2022; Centre for Drug Evaluation and Research, 2024). This study, funded by a European Union (EU) Horizon grant and independent of industry, demonstrated the clinical utility of PGx-guided care. Although the study had an open-label design – a limitation due to the lack of blinding – it had strengths, including a naturalistic, randomised cluster comparator design (Swen et al., 2023). Among patients with DPWG-actionable PGx results, ADRs occurred in only 21.0% of the PGx group compared to 27.7% in the control group, resulting in a significant 30% reduction (Swen et al., 2023). When expanded to include all patients, this reduction remained significant (21.5% in PGx vs 28.6% in TAU, *p* < 0.0001), showing a clear benefit of PGx-guided treatment in improving medication safety. Importantly, nearly 45% of the medications used in the trial were psychotropic drugs, further highlighting PGx’s relevance in psychiatric care (Swen et al., 2023).

PGx testing for MDD has the most extensive body of published clinical studies compared to other psychiatric conditions. The most comprehensive meta-analysis by Brown et al. (2022) (10 RCTs and 3 open-label trials, *N* = 4767) found PGx-guided therapy increased remission likelihood by 41% overall and 46% in RCTs alone, with recent umbrella reviews by Tesfamicael et al. corroborating 41–78% higher remission rates compared to TAU (Brown et al., 2022; Tesfamicael et al., 2024). The evidence suggests clinically meaningful benefits but requires careful synthesis due to methodological heterogeneity across studies as outcomes diverge significantly based on study design: industry-sponsored trials and open-label designs reported larger treatment effects than independent, double-blind RCTs, and proprietary methodologies complicate cross-study comparison. The GUIDED trial (industry-sponsored, patient- and rater-blinded, *N* = 1167) reported a 51% relative increase in remission rates with PGx-guided care compared to TAU (Greden et al., 2019). In contrast, the Oslin et al. (2022) non-industry RCT (*N* = 1944), although only raters were blinded, found a 47% relative increase in remission rates (16.5% PGx vs 11.2% TAU) at 12 weeks (Oslin et al., 2022). Unblinded studies like Hall-Flavin et al. reported larger effect sizes (104% relative increase in remission rates) compared to double-blinded RCTs (e.g. GUIDED and GAPP-MDD trials) (Greden et al., 2019; Hall-Flavin et al., 2013; Tiwari et al., 2022). These design factors – potential sponsorship bias, blinding status and panel heterogeneity – all may influence effect sizes, necessitating prioritisation of methodologically more robust meta-analyses and recent independent RCTs when best synthesising evidence for clinical translation.

Although psychotropic PGx research has primarily focused on antidepressant use in adults, interest is expanding to include other treatments such as stimulants for attention-deficit/hyperactivity disorder (ADHD), psychedelic-assisted therapies and psychiatric conditions like autism spectrum disorder (ASD) and schizophrenia. However, across these areas, the evidence supporting PGx-guided treatment in adults remains limited. In adult ADHD, systematic reviews and meta-analyses show that relatively few studies have been conducted, with most yielding inconclusive or negative results (Bonvicini et al., 2016; Contini et al., 2013). While genetic variants affecting enzymes like CYP2D6 and CES1 may influence the metabolism of medications such as methylphenidate and atomoxetine, consistent and clinically actionable findings are lacking (Faraone and Larsson, 2019). Likewise, pharmacogenomic applications in psychedelic-assisted therapies are still emerging, with early data suggesting that variability in drug-metabolising enzymes may impact the pharmacokinetics and subjective effects of substances such as 3,4-Methylenedioxymethamphetamine (﻿MDMA), psilocybin and D-lysergic acid diethylamide (LSD) (Halman et al., 2025). Yet, this research remains preliminary. In ASD, PGx studies in adults are scarce, as most research has been conducted in paediatric or mixed-age populations (Yoshida et al., 2021). Although some observational studies reported improved outcomes with PGx-guided prescribing in mixed-age cohorts of ASD, robust evidence in adults is still lacking (Arranz et al., 2022). Similarly, in schizophrenia, a single-blind RCT conducted in Denmark with 311 adults diagnosed with schizophrenia found no significant improvements in drug tolerability or effectiveness when PGx testing guided antipsychotic treatment compared to TAU (Jürgens et al., 2020). Across psychiatric conditions, studies involving mixed adult populations have produced variable results – some indicating benefits in depression and anxiety (Papastergiou et al., 2021; Zastrozhin et al., 2020), while others found no significant differences in outcomes (King et al., 2020). Collectively, these findings highlight the novelty and evolving nature of PGx applications beyond antidepressants and underscore the need for larger, well-powered studies focused on clinically meaningful outcomes in adult psychiatric populations.

## Health economic implications

While clinical benefits provide compelling evidence for PGx adoption, widespread implementation in resource-constrained healthcare systems ultimately depends on demonstrating economic feasibility. A 2022 Italian study found CYP2D6/CYP2C19 testing for adult patients with MDD cost-effective at €75,000 per quality-adjusted life year threshold (Carta et al., 2022). A Canadian study in 2023 found that PGx-guided treatment in adult patients with moderate–severe MDD was predicted to an average cost saving of $4926 per patient over a 20-year period (Ghanbarian et al., 2023). This finding aligns with Abushanab et al. (2024), who reported Qatari Riyal (QAR) 2289 in health system savings from PGx-guided therapy for adults with moderate to severe MDD in Qatar (Abushanab et al., 2024). Building on this evidence, a 2024 study by Skokou et al. analysed the economic impact of psychotropic PGx using data from the 2023 ‘mega trial’ by Swen et al. (Skokou et al., 2024). Funded by the EU Horizon 2020 programme, this study focused on assessing the health economic utility of PGx-guided treatment, conducting a cost-benefit analysis that included ADRs, hospitalisations, readmissions and polypharmacy (Skokou et al., 2024). The cohort included 1076 adult psychiatric patients with schizophrenia, MDD or bipolar disorder, drawn from the larger cohort.

Skokou et al. found that patients receiving PGx-guided care experienced 34.1% fewer ADRs, 41.2% fewer hospitalisations and 40.5% fewer readmissions (Skokou et al., 2024). Hospitalisation duration was also reduced (3305 total days in the PGx group vs 6517 in the TAU group), as was re-hospitalisation time (579 vs 928 days). Polypharmacy rates were lower in the PGx group, and mortality was reduced (1 death vs 9 in the TAU group) (Skokou et al., 2024). Economically, there was a 48.5% reduction in treatment costs in the PGx group, making a compelling case for the adoption of PGx in psychiatric care.

These results may not fully apply to primary care, where patients are generally less acute. In this setting, the health economics of PGx should consider test costs relative to indirect expenses, such as lost work time due to slower recovery or trial-and-error medication expenses. Further cost-benefit studies are recommended to clarify PGx’s economic value in primary care. While some studies, including a Markov model comparing CYP2D6 screening to standard care, have explored PGx’s utility, results indicate significant uncertainty around cost-effectiveness, emphasising the need for more research to resolve these uncertainties (Sluiter et al., 2019).

## Implementation challenges in Australia

While clinical utility and cost-effectiveness are critical for PGx adoption, they remain insufficient without systematically addressing implementation barriers. A major obstacle in Australia is the significant knowledge gap, which spans healthcare education, the development and use of clinical guidelines and research that reflects the needs and diversity of the local population. While 68% of Australian medical schools include PGx content, only 15% address clinical implementation, with minimal focus on patient communication or multidisciplinary collaboration (Thomas et al., 2024). Surveys by Pearce et al. (2022) across three hospitals in Sydney reported that only 13% of clinicians felt confident ordering PGx tests, and just 15% expressed confidence in effectively interpreting the results (Pearce et al., 2022). This persists despite clinicians expressing generally positive views about the perceived clinical utility of pharmacogenomic testing in their practice (Ewasiuk et al., 2024). Continuing professional development opportunities specific to PGx are scarce, and existing resources are often not tailored to the needs of busy clinicians (Ewasiuk et al., 2024).

The absence of clear, nationally endorsed clinical guidelines for when and how to use PGx testing in Australia is also significant barrier. While international guidelines exist, they are not always directly applicable due to differences in drug availability and population genetics (Ewasiuk et al., 2024). In response, the Royal College of Pathologists of Australasia (RCPA) has recently made important strides by developing evidence-based guidance for PGx testing of 35 commonly used medications (RCPA, 2024). This initiative has adapted international evidence to the Australian context by considering local drug availability and prescribing practices. The Royal Australian and New Zealand College of Psychiatrists (RANZCP) has endorsed the RCPA’s guidelines, recognising their relevance and utility in supporting clinical decision-making in psychiatry (RANZCP, 2024). However, important gaps remain: The current guidance covers only a fraction of medications with known gene-drug interactions, and uptake is limited by challenges such as a lack of integration into clinical workflows, insufficient clinician education and limited Medicare funding for most PGx tests (Ewasiuk et al., 2024). In addition, the guidelines may not fully address the genetic diversity of Australia’s multicultural and Indigenous populations given the majority of existing PGx evidence is derived from European populations (*Pharmacogenomics Incubator Project Working Group, 2022*; RCPA, 2024)

## Future directions

While current PGx guidelines – such as those from CPIC, DPWG and the FDA – primarily focus on single drug-gene pairs, emerging literature suggests that a pharmacokinetic polygene pathway approach could yield greater clinical and economic advantages. This strategy, often referred to as ‘combinatorial’ PGx, involves considering multiple genes simultaneously. However, combinatorial PGx approaches also face unique challenges. Unlike single-gene CPIC/DPWG guidelines, many commercial combinatorial tests rely on proprietary algorithms that weigh genetic contributions without disclosing their decision matrices (so-called ‘black box’ models) (Nguyen et al., 2024). This lack of transparency has been linked to deviations from evidence-based prescribing of antidepressants when using colour-coded ‘traffic light’ binning system (Nguyen et al., 2024). Standardised tools like Sequence2Script help mitigate this risk by providing open-access, CPIC-aligned recommendations based on individual genotypes and concomitant medications (Nguyen et al., 2024).

Early research proposed that blood-brain barrier (BBB) polymorphisms such as ABCB1 might influence psychotropic dosing, but recent meta-analyses indicate minimal standalone clinical impact (e.g. odds ratio 1.3 for ABCB1 rs1128503) (Breitenstein et al., 2015; Magarbeh et al., 2023). The next generation of psychotropic PGx may involve combining BBB polymorphisms with hepatic phase I metabolism variants to better personalise medication dosages. Although the literature on this approach is sparse, both CGAS and RCT data suggest its potential (Bousman et al., 2019). This pharmacokinetic polygene pathway strategy could offer greater clinical utility for psychotropic PGx (Skvarc et al., 2024). Future advancements may also include epigenetic markers (e.g. BDNF methylation status) or monoamine pathway polymorphisms, such as the serotonin transporter (SLC6A4), to further enhance psychotropic PGx (Hack et al., 2019; Niitsu et al., 2013; Webb et al., 2020).

Criticism of the available evidence being mainly industry-funded has been raised due to the potential conflicts of interest, skewing priorities towards commercially viable areas and limiting data transparency (Brown et al., 2020; Forester et al., 2020; Jablonski et al., 2020; Winner and Dechairo, 2015; Winner et al., 2015). To address these concerns, strategies such as independent oversight, public-private partnerships, diversifying funding, open data initiatives and standardised reporting can promote more objective and ethical research (*Pharmacogenomics Incubator Project Working Group, 2022*).

## Conclusion

In conclusion, recent data from 2023 provide robust evidence supporting both the clinical and economic utility of psychotropic PGx, particularly for antidepressant drug-gene pairs involving CYP2D6/CYP2C19. While concerns remain about commercial bias and limited industry-independent studies, especially in areas beyond antidepressants, psychotropic PGx offers a cost-effective solution amid rising prescriptions and healthcare budget pressures. As guidelines mature and healthcare payers look to reduce costs, broader adoption of psychotropic PGx appears increasingly likely in the near future. Further primary care research is recommended given general practitioners’ major role in mental health consultations (Australian Bureau of Statistics 2024). Advancing PGx implementation through practitioner education and test reimbursement policies is also essential (Polasek et al., 2019).
